# Editorial: Biomedical image or genomic data characterization and radiogenomic/image-omics

**DOI:** 10.3389/fgene.2022.994880

**Published:** 2022-08-17

**Authors:** Ming Fan, Jiangning Song, Zhaowen Qiu

**Affiliations:** ^1^ Institute of Biomedical Engineering and Instrumentation, Hangzhou Dianzi University, Hangzhou, China; ^2^ Department of Biochemistry and Molecular Biology, Biomedicine Discovery Institute, Monash University, Melbourne, VIC, Australia; ^3^ Institute of information Computer Engineering, Northeast Forestry University, Harbin, China

**Keywords:** radiomics, radiogenomics, image-omics, medical imaging, feature analysis, precision medicine, biomarker identification

Precision medicine has emerged as a practical solution for disease care thanks to advances in high-throughput data generation and analysis. Much of the emphasis in discussions about precision medicine or personalized medicine has been focused on the molecular characterization of tissues. However, as genetics differ between and within tumors and are quite heterogeneous, molecular characterizations are limited. Furthermore, there is no easy methodology yet to unravel why tumors with similar characteristics respond differently to a targeted therapy.

Imaging is relatively noninvasive and is often used in routine clinical practice for disease diagnosis, treatment, and prognosis. Medical imaging can provide a comprehensive view of entire tumor lesions; it is commonly used in clinical practice to monitor the progress of the cancer during treatment. The imaging includes but is not limited to ultrasound, X-ray, computed tomography (CT), magnetic resonance imaging (MRI), and positron emission tomography (PET).

Radiomics refers to the conversion of images to high-dimensional data and subsequent mining for the characterization of biology and ultimately to improve disease management for patients. Radiogenomics investigates relationships between imaging features and genomics, which represents the correlation between the anatomical-histological level and the genomic level.

With advanced artificial intelligence methods, especially deep learning, data processing, feature extraction and data integration, medical image- or genomic data-based precision medicine has been greatly improved. There are 15 papers in this Research Topic: “*Biomedical image or genomic data characterization and radiogenomic/image-omics*.” The articles focus on machine learning methods-based biomarker identification from genomics or biomedical imaging to predict disease diagnosis, treatment, and prognosis.

For genomic-based biomarkers in precision medicine, we include nine papers focused on identifying molecular signatures by proposing machine learning algorithms in precise disease diagnosis and treatment management. Machine learning methods, such as gene‒gene interactions and classification/regression models, have been developed to identify diagnostic/prognosis biomarkers.

We present one paper on building gene signatures in cancer prognostic analysis. Liang et al. established a ferroptosis-related gene-based prognostic model to investigate the prognosis of lung adenocarcinoma. Seven ferroptosis-related genes (FRGs) with prognostic significance were identified for dividing patients with lung adenocarcinoma into high-risk and low-risk groups. The results demonstrate the prognostic significance of FRGs in patients with lung adenocarcinoma, which may regulate tumor progression through a variety of pathways.

We also present two papers that employ a gene‒gene interaction-based machine learning algorithm in predicting disease statuses. Cope et al. proposed a machine learning algorithm that use large amounts of data to find gene‒gene interactions that they showed outperformed a polygenic risk score for predicting Parkinson’s disease status. This work advances the state of art in prediction of susceptibility to complex traits or diseases.

Liu et al. established oncogene Aurora kinase A (AURKA)-related gene signatures for predicting the prognosis of patients with hepatocellular carcinoma (HCC) by a protein‒protein interaction network analysis. Eight AURKA-related genes were thus identified that can effectively stratify the risk of HCC patients with differing survival rates. Additionally, patients in the high-risk group showed a higher percentage of immune cell infiltration and higher immune checkpoints. The identified gene signatures can be used as a candidate prognostic marker and therapeutic target in patients with HCC.

We also present one paper that analyzes gene pathways in predicting treatment response. Dai et al. investigated the roles of the Keap1-Nrf2 and PI3K-Akt pathways in esophageal squamous cell carcinoma (ESCC) treated with chemoradiotherapy. The results demonstrate that patients with dysregulated PI3K-Akt pathway exhibit a better survival outcome than patients with an intact PI3K-Akt pathway. This study highlighted the prognostic implications of aberrant cancer pathways in ESCC patients, which may be valuable in guidance of chemoradiotherapy management and treatment-induced toxicity.

We include one paper identifying lncRNA biomarkers for cancer survival analysis. Li et al. explored the biological functions and prognostic significance of epithelial-mesenchymal transition (EMT)-related lncRNAs in patients with colorectal cancer (CRC). A clinical factors and risk signature-based predictive nomogram was established for survival analysis. This signature was verified by predicting the immune-related phenotype and was found to be associated with immune cell infiltration and tumor mutation burden. This study indicated the clinical significance of the identified 11-EMT-lncRNA signature in predicting survival and immunotherapeutic response in CRC.

We also include papers analyzing immune subtypes in disease management. Yu et al. identified three immune-related subgroups for predicting immune checkpoint blockade (ICB) therapy response in lung adenocarcinoma (LUAD). The immune subgroup with higher infiltration scores exhibited a good response to IBC therapy and a better survival, whereas the subgroup with lower scores for immune checkpoint-related genes but higher infiltration scores for suppressive immune cells is more likey to be resistance to ICB therapy and have a poor prognosis. The identified immune subgroup can be promising in preoperatively discriminating LUAD patients with differing ICB therapy responses for a better guidance in treatment management.

Zhang et al. investigated the clinical implications of biglycan in gastric cancer prognosis. They identified biglycan-related differentially expressed genes (DEGs) by comparing the expression of biglycan in gastric cancer and normal tissues. The differential expression was verified through real-time PCR and immunohistochemistry. The constructed nomogram can accurately predict the survival outcomes of patients with gastric cancer. This study demonstrates that biglycan may be important in the occurrence and progression of gastric cancer.

Chen et al. identified human myometrial transcriptome and established the Competing endogenous RNA (ceRNA) regulatory network depending on labor duration. This study highlights the roles of dynamic changes that occur at ceRNAs during parturition in functional changes in human myometrium at labor.

We also included one study that used multiomics biomarkers in disease prognosis analysis. Xiao et al. aims to reveal the prognostic nontumor cell landscape in glioblastoma niches by a multiomics analysis. The biomarkers of nonmalignant cells in the microenvironment of glioblastoma multiforme (GBM) were identified, which separate patients into negative or positive immune response clusters with significantly different survival rates. Negative immune response markers were particularly enriched.

We included six radiological image-based studies in predicting tumor status, survival outcomes, metastases and treatment response. Quantitative mining of data from radiological images, including MRI, ultrasound and CT, was performed, with applications in precise disease diagnosis and prognosis analyses.

We include one paper using radiomics extracted from ultrasound for the diagnosis of ovarian neoplasms. Qi et al. established a nomogram integrating ultrasound-based radiomics signatures and clinical factors, named combined clinical-radiomics (CCR), to discriminate between benign, borderline, and malignant serous ovarian tumors. This CCR-based model shows better prediction performance than a clinical factor-based model.

We present two papers on disease treatment response prediction based on radiomic signatures. Xia et al. developed and validated a nomogram integrating radiomics, MRI findings, and clinicopathological factors to predict survival in triple-negative breast cancer patients treated with neoadjuvant chemotherapy. The proposed signatures significantly stratified patients into high- and low-risk groups with different survival rates. These signatures were further validated in an external validation group. Three indicators, including the multifocal/centric disease status, pathological complete response status, and Rad-score, were independently associated with survival. The results demonstrated that the integrated signature-based nomogram improved the accuracy of survival prediction.

Wang et al. identified MRI features for predicting antipsychotic medication treatment outcomes in schizophrenia. To this end, nine categories of MRI measures and the polygenic risk score (PRS) were combined to separate the responders and nonresponders. The results showed that the PRS was better in prediction performance than measures of cortical thickness, cortical volume, and surface sulcal depth but lower than GMV, ALFF, and surface curvature.

We include one paper on brain metastasis prediction using imaging features derived from CT. Wang et al. identified pretreatment thoracic CT biomarkers for predicting brain metastases in patients with ALK-rearranged non-small cell lung cancer (NSCLC). A machine learning method was proposed to identify the radiomic features extracted from pretreatment thoracic CT images, which achieved good performance in predicting brain metastases within 1 year after detection of the primary tumor.

Wang and Li performed a unified framework for estimating inattention, which is one of the most useful clinical symptoms in attention deficit hyperactivity disorder (ADHD). To improve the classical brain-behavior models, the phase synchrony features were identified from resting state functional MRI (fMRI) using a machine learning method. Among the brain networks, the bilateral subcortical-cerebellum networks exhibit the most predictive phase synchrony patterns for inattention estimation.

We thank the authors and the reviewers for their contribution to this Research Topic. This Research Topic of articles may serve as an inspiring compendium for future research in biomedical imaging or genomic data characterization and radiogenomic/image-omics.

